# The Role of Organizational Policies and Protocols in Service Providers’ Delivery of Appropriate Services to Sex Trafficked Persons in Canada

**DOI:** 10.34172/ijhpm.8869

**Published:** 2026-05-17

**Authors:** Janice Du Mont, Tonia Forte, Sarah Daisy Kosa, Sheila Macdonald, Robin Mason

**Affiliations:** ^1^Women’s College Hospital Research & Innovation Institute, Women’s College Hospital, Toronto, ON, Canada.; ^2^Dalla Lana School of Public Health, University of Toronto, Toronto, ON, Canada.; ^3^Ontario Network of Sexual Assault/Domestic Violence Treatment Centres, Toronto, ON, Canada.

**Keywords:** Sex Trafficking, Commercial Sexual Exploitation, Service Providers, Protocol, Policy

## Abstract

Organizational policies or protocols have been recommended as a potential means of improving sex trafficking services. Therefore, we examined the role of organizational policies or protocols on service providers’ perceptions of challenges in responding to sex trafficked persons. Data were collected using an online, anonymous, national survey between February and August 2023. The healthcare, social, and community service providers surveyed were asked to what extent they agreed or disagreed with the statements, "There are challenges that prevent me from providing the appropriate care, support, or services to sex trafficked persons" and "There are organizational policies or protocols in my place of work that provide guidance on how to respond to sex trafficked persons." The analysis included 553 respondents, of whom almost three quarters (72.6%) perceived challenges and less than half (44.9%) reported the availability of organizational policies or protocols. Respondents who worked in an organization with policies or protocols were less likely than those who did not to report challenges in responding to sex trafficked persons (40.9% vs. 55.3%, unadjusted odds ratio=0.56, *P*=.003; adjusted odds ratio=0.64, 95% CI=0.42, 0.96, *P*=.03). Our findings highlight the importance of implementing organizational policies or protocols that provide guidance on and facilitate the delivery of appropriate care, support, and services to sex trafficked persons. This may better position service providers to address the serious physical, sexual, and mental health sequalae sex trafficked persons experience.

## Background

 Persons who are trafficked, either for forced labour and/or sexual exploitation, experience a range of serious physical, sexual, and mental health sequelae.^1-4^ As those who are trafficked may seek supports during their exploitation, healthcare, social, and community service providers are ideally situated to identify and provide essential care to trafficked persons.^4^ Such service providers, however, have reported that they feel unprepared to identify and respond to the diverse and complex needs of those being trafficked.^4-12^ Factors that may limit their ability to deliver optimal care and support to trafficked persons include a lack of education and training in trauma-informed, culturally sensitive care and knowledge on available local community resources.^4,6,7,10,12^

 Organizational policies and protocols can potentially facilitate the delivery of effective and appropriate care to trafficked persons by providing guidance and direction on how to identify key indicators of trafficking, integrate a trauma-informed approach to care, and provide trafficked persons with helpful community resources and referrals.^4,5,10,13,14^ Indeed, previous studies have found that the lack of appropriate organizational protocols on how to address trafficked persons is among the most common barriers to providing optimal care.^4,5,10^ Organizational policies and protocols, particularly those that include reference to multidisciplinary collaboration, have been recommended as a potential tool for mitigating some of the barriers identified in previous research.^4,7,10,13^

 In Canada, recent research focusing on sex trafficking specifically has examined key organizational and provider-related challenges in caring for sex trafficked persons.^5-9^ To add to this nascent body of work, we examined the role of organizational policies or protocols on service providers’ perceptions of challenges in providing appropriate care, support, or services to sex trafficked persons. The findings of this study may inform future initiatives to better position service providers to address the serious physical, sexual, and mental health sequalae sex trafficked persons experience.

## Methods

###  Design 

 An anonymous, online, cross-sectional survey was conducted between February and August 2023. A recruitment flyer was prepared and posted on social media platforms (Facebook, Twitter/X, and Instagram) and was distributed via email among the research team’s professional networks and professional associations and organizations across the country to share with their membership.

 The flyer contained a brief description of the study, a declaration of anonymity, and an offer of a $25 e-gift card for participation. The flyer also instructed those interested in participating in the study to contact the project coordinator via email. To prevent automated bot responses to the survey, those expressing an interest in participating were verified by the project coordinator who asked potential respondents to provide their name and organization. They were then sent an individualized link to an online form which confirmed their eligibility (ie, working in Canada in healthcare/health services, social/community services, the education sector, or police services, able to read and write in English, and able to provide informed consent).

 The survey captured perceptions of, and capacity to respond to, sex trafficking, sociodemographic information including geographic location, work-related experience, and training and expertise on sex trafficking.

 The study methodology and survey instrument are described in more detail in Forte et al.^15^

###  Variables

 For this study, the outcome variable of interest was measured by the survey question, “There are challenges that prevent me from providing the appropriate care/support/services to sex trafficked persons.” Responses were captured on a 6-point Likert scale (strongly disagree to strongly agree).

 Independent variables of interest included age group (18-24, 25-34, 35-49, 50-64, 65+, Prefer not to answer), gender identity (Female/Woman, Male/Man, Nonbinary, Questioning/Exploring, Prefer not to respond/Prefer not to disclose, Gender identity not listed, Please specify), assigned gender at birth (Female, Male, X, Unsure, Prefer not to respond/Prefer not to disclose, Assigned gender at birth not listed), racial/ethnic background as measured by the Government of Ontario Anti-Racism Directorate’s Data Standards for the Identification and Monitoring of Systemic Racism^16^ (White, Black, East/Southeast Asian, Indigenous, Latino, Middle Eastern, South Asian, Another race category, Please specify, Prefer not to answer), highest level of education completed (Less than high school, High school, College, Community college or trade school, Undergraduate degree, Graduate or professional degree, and Other degree not listed, Please specify, Prefer not to answer), province/territory of employment, type of geographic location (Remote community, Rural area [<1000 people], Small town [1000 and 29 999 people], Medium-sized city [30 000 and 99 999 people], Large city [100 000 and 499 999 people], Very large city [500 000+ people]), Primary field of work (Healthcare/health services/supports, Social/community services/supports, Education sector, Police services), years in field of work: (0-5 years, 6-10 years, 11-15 years, 16-20 years, 21+ years), and previous experience working with sex trafficked person(s) in current position (Yes, No).

 Education and/or training on sex trafficking was measured by the question, “What education or training (in-person or online) on sex trafficking have you had? Select all that apply” (Academic class, Academic course, Professional training program, Self-directed learning, Workshops, Webinars [eg, from a community-based organization], Culturally specific programming [eg, Indigenous-led], Conferences, Community of practice, Media/social media [please specify], Other [please specify], and None). Respondents who indicated having had received training via any of the methods listed were categorized as having received training and those who selected “None” were categorized as having received no training. Level of expertise related to sex trafficking was measured by the survey question, “How would you rate your current level of expertise with regard to responding to those who are being or have been sex trafficked?” Response categories included: No expertise, Low level of expertise, Moderate level of expertise, High level of expertise, and Very high level of expertise.

 To determine respondents’ perceived support from their organization to respond to sex trafficking, or if their place of work had internal policies or protocols to address the specific needs of sex trafficked persons, respondents were asked to respond to: “I have the support I need from my organization to respond to someone having been or being sex trafficked” and “There are organizational policies or protocols in my place of work that provide guidance on how to respond to sex trafficked persons.” For these items, a 6-point Likert scale (“Strongly disagree” to “Strongly agree”) was used to quantify respondents’ level of agreement.

###  Statistical Analysis 

 As healthcare/health services and social/community services can be similar in their approach when providing supportive care to sex trafficked persons, we limited our analysis to those respondents who indicated their field of work to be in these sectors. A “gender” variable was computed based on methods described by Kronk et al^17^ using responses to survey items on gender identity and assigned gender at birth.

 For some variables, original groupings were recategorized for the bivariate and multivariate analyses to adjust for small cell sizes. Province of employment was re-categorized into region of employment as follows: (West [British Columbia], Prairie [Alberta, Saskatchewan, Manitoba], Central [Ontario, Quebec], Atlantic [New Brunswick, Nova Scotia, Prince Edward Island, Newfoundland and Labrador], North [Yukon Territory, the Northwest Territories, and Nunavut]). Age was regrouped as 18-34 years, 35-49 years and 50+ years. Racial/ethnic background was re-categorized as White or Another race/ethnicity. Highest level of education completed was grouped as Undergraduate degree or less vs. a Graduate or Professional degree. Type of geographic location was re-grouped as Remote or Rural or Small town vs. Medium-sized city vs. Large or Very large city. Level of expertise in responding to sex trafficking was re-grouped as No expertise vs. Low level of expertise vs. Moderate level of expertise vs. High or Very High level of expertise. Finally, years in field of work was re-grouped as 0-5 years, 6-15 years, and 16+ years.

 For all variables, the amount of missing data was ≤1%, and these cases were excluded from the analyses.

 The outcome variable, challenges in providing appropriate care/support/services, and the organizational policies and protocols variable were dichotomized with “Slightly disagree,” “Disagree,” and “Strongly disagree” grouped and “Slightly agree,” “Agree,” and “Strongly agree” grouped. Bivariate associations between the outcome variable and independent variables were assessed using the Pearson chi-square test.

 To determine the factors associated with perceived challenges in providing appropriate supports to sex trafficked persons, we conducted a multivariate logistic regression analysis. Variables that were associated with perceived challenges in the bivariate analysis at *P* < .10 were included in the model. Variables were checked for multicollinearity.^18^ Variables that did not contribute independently to the outcome at *P* < .05 following multivariate adjustment were removed from the final model. To determine the impact of dichotomizing the outcome variable, a sensitivity analysis was conducted using ordered logit regression analysis which preserved the Likert-scale responses.

## Results

 A total of 775 individuals indicated an interest in participating in the survey and were sent an eligibility questionnaire. Of these, 602 met the eligibility criteria and completed at least one question in the online survey. Of these, forty-nine individuals were excluded from the present analysis as they indicated their field of work to be education (n = 26) or police services (n = 23). The final analytic sample included 553 healthcare, social, and community service providers.

 Eighty-eight percent of the survey sample were cisgender female/woman and approximately two in five (41.3%) were aged 35-49. Most respondents indicated their race or ethnic background to be White (77.0%), worked in Central Canada (70.7%), and worked in a large or very large city (62.2%). Most were university educated (41.4% reported completing an undergraduate degree and 43.4% reported a graduate or professional degree).

 Two thirds (65.1%) of respondents reported having worked with a person who had been or is currently being sex trafficked and most (86.8%) received some form of education and/or training in sex trafficking. When asked to rate their level of expertise in responding to sex trafficked person(s), most rated their level of expertise as low (36.4%) or moderate (32.1%).

 Almost three quarters (72.6%) of respondents agreed that challenges prevent them from providing the appropriate care, support, or services to sex trafficked persons. Less than half of those surveyed (44.9%) reported that their organization had policies or protocols in place that provide guidance on how to respond to sex trafficked persons.

Table 1 shows the bivariate associations of factors associated with perceived challenges in providing the appropriate care, support, or services to sex trafficked persons.Variables associated with the outcome at *P* < .10 (region of employment, education and/or training on sex trafficking, level of expertise in sex trafficking and working at an organization with policies or protocols in place to respond to sex trafficked persons) were entered into the multivariate logistic regression model. Perceived support from organization to respond to sex trafficking was collinear with the policies and protocols variable and, therefore, was not included in the final multivariate model.

**Table 1:** Bivariate Analysis of Factors Associated With Perceived Challenges in Providing Appropriate Care to Sex Trafficked Persons Among 553 Canadian Healthcare, Social, and Community Service Providers

	**Challenges Prevent Me From Providing the Appropriate Care,** **Support, or Services to Sex Trafficked Persons**		***P*** ** Value**
	**Agree**	**Disagree**	
	** No. (%)**	** No. (%)**	
Age group (y)			
18-34	151 (38.0)	60 (40.0)	.46
35-49	170 (42.8)	56 (37.3)	
50+	76 (19.1)	34 (22.7)	
Gender			
Cisgender female; Woman	344 (87.1)	136 (90.7)	.34
Cisgender Male; Man	18 (4.6)	7 (4.7)	
Transgender and gender diverse	33 (8.3)	7 (4.7)	
Racial/ethnic background			
White	308 (78.4)	109 (73.1)	.20
Another race/ethnicity	85 (21.6)	40 (26.9)	
Highest level of education completed			
Undergraduate or less	218 (54.9)	91 (61.1)	.20
Graduate/professional degree	179 (45.1)	58 (38.9)	
Region of employment			
West	31 (7.8)	4 (2.7)	.03
Prairie	60 (15.1)	19 (12.7)	
Central	269 (67.6)	117 (78.0)	
Atlantic	30 (7.5)	5 (3.3)	
North	8 (2.0)	5 (3.3)	
Type of geographic location			
Remote/rural/small town	78 (19.6)	29 (19.3)	.95
Medium-sized town	72 (18.1)	29 (19.3)	
Large/very large city	248 (62.3)	92 (61.3)	
Field of work			
Healthcare services/supports	193 (48.5)	71 (47.3)	.81
Social/community services/supports	205 (51.5)	79 (52.7)	
Years in field of work			
0-5	91 (22.9)	40 (26.7)	.65
6-15	177 (44.5)	63 (42.0)	
16+	130 (32.7)	47 (31.3)	
Experience working with sex trafficked person(s)			
No	144 (36.2)	47 (31.3)	.29
Yes	254 (63.8)	103 (68.7)	
Education and/or training on sex trafficking			
No	58 (14.6)	13 (8.7)	.07
Yes	340 (85.4)	137 (91.3)	
Level of expertise in responding to sex trafficking			
None	79 (19.9)	18 (12.0)	.02
Low	150 (37.7)	50 (33.3)	
Moderate	114 (28.6)	62 (41.3)	
High/very high	55 (13.8)	20 (13.3)	
There are organizational policies or protocols in my place of work that provide guidance on how to respond to sex trafficked persons			.003
No	235 (59.1)	67 (44.7)	
Yes	163 (40.9)	83 (55.3)	
I have the support I need from my organization to respond to someone having been or being sex trafficked			<.001
No	172 (43.2)	31 (20.7)	
Yes	226 (56.8)	119 (79.3)	

 The multivariate logistic regression analysis (Table 2) showed that healthcare, social, and community service providers who worked in an organization with policies or protocols in place that provide guidance on how to respond to sex trafficked persons were less likely to perceive challenges in providing appropriate care, support, or services to sex trafficked persons (Adjusted odds ratio = 0.64, 95% CI = 0.42, 0.96, *P* = .03). We also found that those working in Western Canada were more likely to perceive challenges in providing care compared to those employed in Central Canada (Adjusted odds ratio = 3.18, 95% CI = 1.09, 9.32, *P* = .04). Respondents who reported a moderate level of expertise in responding to those who are being/have been sex trafficked were less likely to perceive challenges in responding compared to those who reported no expertise (Adjusted odds ratio = 0.50, 95% CI = 0.25, 1.0, *P* = .049).

**Table 2:** Logistic Regression Analysis of Factors Associated With Perceived Challenges in Providing Appropriate Care to Sex Trafficked Persons Among Canadian Healthcare, Social, and Community Service Providers

**Variable**	**Unadjusted Odds Ratio**	**Multivariate Model**		
		** Adjusted Odds Ratio**	**95% Confidence Interval**	***P*** ** Value**
Region of employment				
Central	Reference			
West	3.38	3.18	(1.09, 9.32)	.04
Prairie	1.37	1.14	(0.63, 2.03)	.67
Atlantic	2.61	2.36	(0.88, 6.34)	.09
North	0.70	0.61	(0.19, 1.94)	.40
Education and/or training on sex trafficking				
No	Reference			
Yes	0.56	0.90	(0.43, 1.87)	.77
Self-rated level of expertise in responding to sex trafficking				
None	Reference			
Low	0.68	0.73	(0.38, 1.41)	.35
Moderate	0.42	0.50	(0.25, 1.00)	.049
High/very high	0.63	0.90	(0.39, 2.05)	.80
There are organizational policies or protocols in my place of work that provide guidance on how to respond to sex trafficked persons				
No	Reference			
Yes	0.56	0.64	(0.42, 0.96)	.03

 The findings from the sensitivity analysis using ordered logit regression were consistent with those from the logistic regression analysis and showed that respondents working at an organization with policies or protocols in place that provide guidance on how to respond to sex trafficked persons were significantly less likely to perceive challenges in providing appropriate care (Adjusted odds ratio = 0.62, *P* = .005).

## Discussion

 Most healthcare, social, and community service providers in our national sample reported having worked with a person who had been or was currently being sex trafficked and received some form of education and/or training in sex trafficking. Despite this, almost three-quarters of providers in our sample perceived challenges in providing the appropriate care, support, or services to sex trafficked persons. Our results are consistent with previous work showing that education and training alone are not enough for healthcare, social, and community service providers to feel confident in responding to sex trafficked persons.^5^

 One strategy, which has been identified/recommended in previous research for mitigating some of the barriers to providing optimal care, is the implementation of institutional or organizational level policies or protocols that provide guidance on how to respond to sex trafficked persons.^4,5,10,19,20^ The HEAL Trafficking Protocol Toolkit for Developing a Response to Human Trafficking Victims in Health Care Settings provides an example of a framework that can be used to create a protocol for identifying and responding to trafficked persons, adaptable to various health settings.^20^ In our study, providers working at organizations that had policies or protocols in place that provide guidance on how to respond to sex trafficked persons were less likely to perceive challenges in providing appropriate care, support, or services to this population, suggesting that the development and implementation of such institutional policies should be a priority in service settings where providers are most likely to encounter those being sex trafficked. Correctly understanding and applying policies and protocols will be crucial for effectively supporting sex trafficked persons.

 While organizational policies and protocols that provide guidance on how to respond to sex trafficked persons are clearly important, in our sample, less than half of all providers surveyed reported that their organization had such policies or protocols in place. These results are consistent with US based studies that have assessed the readiness of healthcare institutions to respond to trafficking and found that few had policies or protocols in place to screen for trafficked persons.^19,21,22^ Among those that did, the content of the protocols sometimes varied widely across organizations.^19^ In the Canadian context, qualitative studies have begun to document the specific challenges faced by healthcare and social service providers when responding to sex trafficked persons^5-7^; results from this research could help inform organizational policies and protocols in Canada and other jurisdictions.

 There are important limitations to this study, most notably, relating to the representativeness of the sample and the generalizability of the findings. First, participation was based on self-selection and, therefore, our sample may be biased toward those respondents who have prior experience or interest in the issue of sex trafficking. Challenges in providing appropriate care to sex trafficked persons may be greater among the general population of healthcare, social, and community service providers without prior experience or awareness related to this issue. Second, while our recruitment strategy included multiple modalities (eg, use of several social media platforms, outreach to professional service associations and organizations from across the country), our sample may not be representative of all service providers in Canada, particularly those who may not access social media or are not part of the professional associations contacted. Third, as we were not able to collect data for those respondents who expressed an interest in participating but did not complete the survey, we were unable to examine how these non-responders differed from survey respondents. With respect to survey methodology, the nature of the policies and protocols implemented across the different organizations on how to respond to sex trafficked persons were not collected as part of this study and may have varied. Likewise, the concept of “challenges” was not specifically defined in the survey to avoid being prescriptive as to what a respondent might consider an issue in delivering appropriate care and, as a result, may have been interpreted differently by respondents. Future research is needed to determine the specific content in policies or protocols that service providers find most useful to their practice and how such policies or protocols can be effectively implemented and enforced. In addition, while our study focused on sex trafficking specifically, further research should examine policies and protocols that address all forms of human trafficking, as many trafficked persons experience multiple forms of exploitation simultaneously.^23^

 In conclusion, as healthcare, social, and community service providers may encounter trafficked persons in their work, our findings highlight the importance of increasing their capacity and ability to respond effectively. Implementing organizational policies and response protocols that provide guidance on how to respond to trafficked persons can provide significant direction to healthcare, social, and community service providers, facilitating the delivery of optimal care, support, and services.

## Acknowledgements

 We would like to express thanks to our pilot group for their contributions to this project. We thank Mai-Lan Johnston, Rhonelle Bruder, and Frances Montemurro for their contributions to the survey development and Hannah Seo for her contributions to the data collection. We would also like to thank all healthcare, social, and community service providers who responded to the survey.

## Ethical issues

 Ethics approval for this study was received by the Women’s College Hospital Research Ethics Board (REB 2021-0133-E). Respondents provided consent following their review of forms detailing the study and their rights as participants.

## Conflicts of interest

 Authors declare that they have no conflicts of interest.
